# Cold Bathing

**Published:** 1857-11

**Authors:** 


### COLD BATHING

In summer or winter we detest, except it be to jump into a river, splurge about for two or three minutes, and then dress, and walk home as hastily as possible. All animate nature, except the hydric, instinctively shrinks from the application of cold water, if in health. Every body knows that cold water cannot wash the hands clean, and yet whole tomes are scribbled about the purifying effects of cold water. Cold water kills more than it cures. Hundreds of children are killed every year by fanatical mothers sousing them, head and ears, in cold water every day.

We never saw a modern bath-tub until we were thirty years of age, and ever since the sight, we have not ceased to hate it with great cordiality, on account of the mischief which it constantly occasions.

The ordinary use of a bath-tub is an indecency. A great deal of stuff is printed about the bathing habits of the ancients, about the Eastern nations, and their love of the bath. What if they did love it? the ancients have all gone to grass long ago, and "Eastern nations" are going to pot as fast as possible, individually and collectively! The average of human life is shorter, by many years, among the Eastern peoples than among the Western. Of three hundred inhabitants in the United States, only four persons die every year, while six die in England, and eight in France, and the farther we go "East" the greater is the mortality. As to the United States, it is the healthiest country on the globe, as a whole: according to the last statistics, Virginia, the very embodiment of the "*Great Unwashed*," is the healthiest of her healthy sisters, and next comes North Carolina, all smoked with pine knots, and begrimed with coal dust and tar: and it is doubtful if one in ten thousand of its families ever saw a modern bath-tub.

How many of our grandsires, now hale and hearty at three-score-and-ten, ever felt a shower-bath, or jumped into a tub of cold water to wash themselves? Who are they, amongst the beautiful women of present or past time, whose cheeks are the softest, and remain the longest free from the wrinkles of age? They are those who never washed their faces in cold water; and if indeed they were washed at all, it was done with warm water or spirits of wine, as practiced in the times of Louis *Quatorze*. Soft as velvet is the cheek of infancy; and it only grows harsh, and hard, and rough, as the practice gains of washing them with cold water.

A pig gets no cleaner by wallowing in a puddle; yet men and women wallow in a bath-tub, diluting the excretions from nameless parts of the person, to come in contact with the cleaner hands and face, and even lips, it may be!

People talk glibly about the bathing habits of *Eastern Nations* and the cleanliness of the Houris, who grace the Turkish Harem, and then we essay an imitation in this fashion. A Turk takes a hot bath, we take a cold one; we jump into a bath-tub, a thing which no decent Turk ever does. We question if there is a single bath-tub in all the dominions of the Sultan, unless it be the pet property of some water-mad Yankee. A Turk washes himself under a *stream of running water*, after a vigorous first-scrubbing; so that no impure particle, loosened from one part of the body, can, by possibility, come in contact with the body again. We wash ourselves in bath-rooms as cold as Greenland: the Turk cleanses himself in an apartment almost as hot as an oven. We really cannot see how a man can make himself clean in a bath-tub, after the usual fashion.

The sum of the whole matter is this: If we want to cultivate habits of personal cleanliness and health, let us, at rational intervals, say once a week, have a room, *in fire-time*, which shows seventy degrees of Fahrenheit, and with strong soap-suds and a *hog's hair* brush, let the whole body be most thoroughly scrubbed, almost as effectually as if we were rubbing a grease-spot out of a plank-floor, then let the whole surface be rinsed with warm water, *running from the spiggot.* When that is done, an instantaneous souse in a bath-tub, or better still, a bucket of cold water dashed on the head, falling all over the naked person, and then to be wiped dry and dress in two minutes—that indeed is a glorious luxury to any grown person, not an invalid. That "taking a bath" requires the exercise of a sound judgment, and that without this, it is not unattended with fatal consequences, New Yorkers especially have recently had some sad lessons. The lovely young wife of our national representative at Rome went from the dinner-table to a warm bath, and died in a few hours. One of our most distinguished lawyers, the State's Attorney, we believe, was, within a year, found dead in his bath-room. *Mortimer Livingston*, one of New York's noblest merchants, "took a bath one morning, remaining in the water a long time. On coming out, he complained of cold over his entire person, and all the means made use of to restore warmth failed, he lingered awhile, and died in a few days, aged fifty years," in the very prime of life! Bishop Heber, the author of that charming hymn,

"From Greenland's icy mountains," &c.

died from the effects of a bath: and how many thousands of children are annually hurried into the grave by injudicious washings, we will not hazard to conjecture.

Let those who are wise, learn from these things a lesson; and let none controvert the statements made, but those who know something, and can give whole facts.

---

### ABSENCE OF MIND.

To put the candle to bed, and blow yourself out.

